# A phase II study of fixed-dose capecitabine and assessment of predictors of toxicity in patients with advanced/metastatic colorectal cancer

**DOI:** 10.1038/sj.bjc.6603049

**Published:** 2006-03-21

**Authors:** R Sharma, L Rivory, P Beale, S Ong, L Horvath, S J Clarke

**Affiliations:** 1 Sydney Cancer Centre, Sydney, NSW, Australia

**Keywords:** capecitabine, advanced colorectal cancer, efficacy, safety, folate

## Abstract

The purpose of this study was to evaluate the safety and activity of fixed-dose capecitabine in patients with advanced colorectal cancer and to correlate pretreatment plasma concentrations of homocysteine and serum and red cell folate with toxicity. Patients received capecitabine 2000 mg (4 × 500 mg tablets) twice daily on days 1–14 every 3 weeks. They were reviewed weekly during the first cycle and then three weekly for safety assessment. Eligibility criteria were advanced/metastatic colorectal cancer, ⩽2 prior chemotherapy regimens, ECOG performance status 0–2 and life expectancy >12 weeks. A total of 60 patients were enrolled and 55 were evaluable for efficacy. The median age was 72 years and 63% of patients had a performance status of 1 or 2. Confirmed tumour responses were reported in 15 patients (28%; 95% confidence interval (CI), 15.7–40.3%). The median time to disease progression was 4.9 months and median overall survival was 11.2 months. The median ratio of fixed dose to body surface area (BSA)-calculated dose was 88% (range 65–108%). Significant myelosuppression was not observed. Grade 2/3 treatment-related adverse events were diarrhoea (34%), fatigue (27%), stomatitis (15%) and hand–foot syndrome (22%). Dose reduction due to adverse events was required in 16 patients (29%) and multiple reductions in five patients (9%). There was no grade 3/4 haematological toxicity, any grade 4 adverse events or treatment-related deaths. Patients with higher pretreatment levels of serum folate experienced significantly greater toxicity (*P*=0.02, CI: 1.0–1.2) during cycle 1 and over the entire treatment period (*P*=0.04, CI: 1.0–1.3). Pretreatment homocysteine concentrations did not predict for toxicity. In conclusion, fixed-dose capecitabine appears to have similar efficacy and safety compared to the currently recommended dose schedule based on body surface area and simplifies drug administration. A high pretreatment folate may be predictive of increased toxicity from capecitabine.

5-Fluorouracil (5-FU) has been the mainstay of treatment of patients with advanced or metastatic colorectal cancer, and remains an integral component of modern combination regimens. As a single agent, 5-FU improves median survival by approximately 6 months (Midgley and Kerr, 1999). Attempts at producing more effective 5-FU regimens have been made, using continuous 5-FU infusion or biomodulation with agents such as Leucovorin (LV; folinic acid), although improvements in survival have been marginal (Schmoll *et al*, 1999). However, the addition of oxaliplatin and irinotecan to 5-FU has increased response rates and median survival compared to 5-FU alone (de Gramont *et al*, 2000; Douillard *et al*, 2000; Giacchetti *et al*, 2000; Saltz *et al*, 2000). These regimens, however, are associated with increased toxicity and may not be suitable for all colorectal cancer patients. In addition, infusional regimens of 5-FU often require the use of an indwelling central venous catheter, which can be associated with a number of complications, for example, thrombosis, infection, pneumothorax and bleeding. The use of indwelling central venous catheter is also associated with increased hospital resource utilisation (Ardalan and Flores, 1995; Schmoll, 1996; Verso and Agnelli, 2003).

Capecitabine is an oral fluoropyrimidine that preferentially delivers 5-FU to the tumour via a three-step enzymatic conversion, the final step being catalysed by thymidine phosphorylase, which has a higher activity within the tumour compared with healthy tissue (Miwa *et al*, 1998).

The current recommended dose of capecitabine for the treatment of metastatic colorectal cancer is 1250 mg m^−2^ twice daily for 14 days followed by a 7-day rest period. Criticism has been levelled at BSA as a method of calculating doses of cytotoxic agents. Correlation between BSA and physiological functions, such as hepatic and renal function, is weak or nonexistent. Only cardiac output has been shown to correlate with BSA (Sawyer and Ratain, 2001). Gurney (1996) argues for the introduction of a ‘prime’ fixed dose to be determined for each drug. This prime dose would then be modified rationally using known covariates of drug clearance such as renal or hepatic function, or following an initial dose administration based on the extent of toxicity experienced. Capecitabine is available in either 500 or 150 mg tablet size and consequently dosing by BSA can be impractical and the dose is frequently rounded off to a convenient number of tablets. In addition, pharmacokinetic studies demonstrate that when capecitabine doses were determined by BSA, there were large inter-patient variations in the pharmacokinetics of capecitabine and its metabolites (27–89%). This was thought to be primarily due to variability in the activity of metabolic enzymes (Reigner *et al*, 2001). Furthermore, in a bioequivalence study by Cassidy *et al* (1999), no clinically significant effect was observed between BSA and the pharmacokinetics of capecitabine and its metabolites. Therefore, in the current study, we planned to evaluate the efficacy and safety of fixed-dose capecitabine given at 2000 mg (4 × 500 mg tablets) twice daily on days 1–14 every 3 weeks. For instance, an average patient would have a BSA of 1.6 m^2^ that corresponds to a capecitabine dose of 2000 mg daily. This fixed dose was considered to be appropriate for this study.

A number of clinical studies have illustrated that pretreatment levels of folate and homocysteine are predictive for both tumour response and toxicty in patients treated with either thymidylate synthase (TS) inhibitors or folate antagonists (Bunn *et al*, 2001; Calvert, 2002; Niyikiza *et al*, 2002). Increased amounts of exogenous folate leads to enhanced TS inhibition and stability of the ternary complex formed between the enzyme, deoxyuridine monophosphate and the folate cofactor (Pinedo and Peters, 1988). Previous murine studies have indicated that folate levels may predict for the cytotoxic efficacy of 5-FU (van der Wilt *et al*, 2001). Serum and red cell folate and plasma homocysteine each reflect different aspects of a patient's folate status. Therefore, the relationships between folate and homocysteine levels with both response and toxicity following capecitabine treatment were evaluated.

## PATIENTS AND METHODS

### Patients

Patients were eligible for entry into the study if they had locally advanced or metastatic colorectal cancer with measurable or evaluable disease. Patients could have previously received up to two prior chemotherapy regimens for metastatic disease. If administered, adjuvant therapy should have been completed ⩾6 months prior to study entry. Histological or cytological confirmation of colorectal adenocarcinoma was required. Patients had to be at least 18 years of age and have a life expectancy >12 weeks. ECOG performance status (PS) was required to be between 0 and 2. Patients were not included if they had active or extensive brain metastases, active systemic infection, inflammatory bowel disease, unstable cardiac disease or untreated vitamin B12 deficiency. Patients who were pregnant or actively lactating were excluded. Patients had to be able to be changed from warfarin to enoxaparin sodium (Kolesar *et al*, 1999). Patients were not enrolled if initial evaluations revealed significant abnormalities in neutrophils (<1.5 × 10^9^/l), platelets (<100 × 10^9^/l), serum creatinine or serum bilirubin (>1.5 × upper limit of normal), and ALT, AST or alkaline phosphatase (>3 × upper limit of normal).

The study received prior local ethical board approval and was conducted according to the Declaration of Helsinki and its subsequent amendments. All patients gave written informed consent prior to participation in the trial.

### Study treatment

The study was conducted at three different hospitals in New South Wales, Australia, each of which followed the same protocol and was coordinated from a single centre. Capecitabine was administered orally at a fixed dose of 2000 mg twice daily on days 1–14 every 3 weeks. Capecitabine doses were given approximately 12 h apart and taken with water within 30 min following ingestion of food. Treatment with capecitabine was continued until the scheduled assessment at 30 weeks, or until the development of progressive disease or unacceptable toxicity. In responding patients, treatment could be continued beyond 30 weeks at the discretion of the investigator.

### Treatment modifications

Treatment interruption or dose reduction was not indicated for grade 1 toxicity National Cancer Institute Common Toxicity Criteria (CTC) version 2.0 (National Cancer Institute: Common toxicity criteria). Treatment was interrupted in cases of grade 2 toxicity or worse, and was not resumed until toxicity resolved or improved to grade 1. When treatment was resumed, capecitabine doses were reduced as follows: by 25% (1000 mg) for patients who experienced a second occurrence of a given grade 2 nonhaematological toxicity, or a first occurrence of a haematological toxicity or any grade 3 toxicity; or by 50% (2000 mg) for patients who experienced a grade 3 haematological toxicity. Treatment was discontinued if a given toxicity occurred despite dose reduction or for grade 4 toxicity.

### Evaluation of patients

Complete history was recorded, full physical examination performed and blood samples were collected at baseline. Baseline blood analyses included full blood count (FBC), biochemistry, liver function tests (LFTs), vitamin B12, carcinoembryonic antigen (CEA), serum and red cell folate and plasma homocysteine. Serum and red cell folate were measured in a central laboratory using National Committee for Clinical Laboratory Standards (NCCLS) procedures. Serum and red cell folate were measured using a competitive binding receptor assay (Access immunoassay, Beckman and Coulter Inc., Gladesville, NSW, Australia). Samples were not batched, but analysed as patients were enrolled.

Baseline CT imaging of the chest, abdomen and pelvis was obtained within 3 weeks of treatment commencement. At the start of each treatment cycle, history and physical examination were repeated and blood samples were taken for FBC, biochemistry, LFTs and CEA. Patients were reviewed weekly during cycle one and then every 3 weeks for safety assessment. All safety evaluations were graded according to the NCI-CTC except hand–foot syndrome (HFS). Hand–foot syndrome was classified as grade 1 (numbness, dysaesthesia, painless swelling or erythema not disrupting normal activities), grade 2 (painful erythema with swelling or affecting daily living activities) or grade 3 (moist desquamation, ulceration, blistering, severe pain or any symptoms leading to an inability to work or to perform daily living activities) (Blum *et al*, 1999). Tumour response was assessed after every two cycles with repeat CT chest, abdomen and pelvis. Tumour response or progression was recorded using the RECIST criteria (Therasse *et al*, 2000).

### Statistics

For analyses, toxicity was grouped and analysed as grade 0 and 1, grade 2 and 3 or as grade 4. The association between the presence of a serious adverse event (grade 2 or 3) both during cycle 1 and for the whole course of treatment and the following parameters was determined using logistic regression: gender, PS, number of sites of metastases, prior pelvic radiotherapy, creatinine clearance, homocysteine, serum folate, red cell folate, serum vitamin B12 and serum albumin. The odds ratio and its 95% confidence interval (95% CI) were used to quantify the extent of any association. Kaplan–Meier survival curves were used to illustrate the distributions of the time to progression and overall survival.

## RESULTS

### Patients and treatment

A total of 60 patients were enrolled from three centres in Australia between January 2002 and August 2003. Four patients were excluded from analysis as they were not dosed according to protocol and one patient withdrew consent. Therefore, the efficacy and safety analyses included 55 patients.

The demographic and clinical characteristics of the patients are summarised in Table 1. The median age of patients at enrolment was 72 years, and 35 (63%) patients had a performance status of 1 or 2. A total of 13 patients (24%) had received prior adjuvant chemotherapy with 5-FU/LV and two patients (4%) had received prior chemotherapy for metastatic disease (one with oxaliplatin/5-FU and the other with irinotecan/5-FU). Six patients (11%) had received prior pelvic radiotherapy.

### Efficacy

At the time of analysis, 48 patients had died. The cause of death in all patients was disease progression. The best tumour response over the entire treatment period for all treated patients is shown in Table 2. Two patients were not evaluable, as they did not have radiological follow-up. The overall response rate in evaluable patients was 28% (95% CI: 15.7–40.3). In all, 17 patients (32%, 95% CI: 19.2–44.8) had stable disease while the remaining 21 patients (40%, 95% CI: 26.5–53.5) had disease progression. Responses were observed in three (23%) of the patients who had received prior 5-FU-based adjuvant therapy. Median survival was 11.2 months (95% CI: 8.7–13.7) and median time to disease progression was 4.9 months (95% CI: 1.5–8.2).

At the time of analysis, five patients were still receiving capecitabine. The median duration of therapy with capecitabine was 97 days and the median number of cycles received was 6.5. The median dose intensity received was 100% (range 25–114%). The median BSA value of the enrolled patient cohort was 1.8 m^2^. Assuming the normal recommended dose of capecitabine (1250 mg m^−2^ twice daily on days 1–14 every 3 weeks) had been used, the total dose calculated on BSA should have been 63 350 mg during the first cycle of therapy. The median total flat dose received by patients in cycle 1 was 56 000 mg. Therefore, the median ratio of fixed-dose to BSA-calculated dose was 88% (range 65–108%). Thus, patients received a median dose 12% lower than the BSA-calculated dose.

### Safety

The most common treatment-related adverse events are reported in Table 3. Overall, fixed-dose capecitabine was well tolerated with no grade 4 nonhaematological or grade 3/4 haematological adverse events recorded. Fatigue, HFS and diarrhoea were the predominant adverse events reported. Overall, adverse events were reversible and manageable with interruption of dosing and dose reduction. A single dose reduction because of toxicity was required in 16 patients (29%) and multiple reductions were required in five patients (9%). Of the patients that had received prior pelvic radiotherapy, one patient had a 1-week delay in treatment due to grade 3 diarrhoea. The most common adverse event resulting in dose reduction was HFS (22%) and treatment was delayed in 19 (35%) patients. Adverse events led to the cessation of treatment in eight patients (15%). The most frequent adverse event leading to discontinuation was diarrhoea. There were no adverse-event-related deaths during the study.

### Predictors of toxicity

Baseline laboratory test results were not available for all patients (Table 4). The relationship between baseline levels of creatinine clearance, CEA, homocysteine, red cell and serum folate, serum vitamin B12 and albumin and toxicity during cycle 1, and for the whole treatment course were assessed. No significant relationship was seen between toxicity and creatinine clearance (*P*=0.8), CEA (*P*=0.5), homocysteine (*P*=0.2), red cell folate (*P*=0.7), serum vitamin B12 (*P*=0.3) and albumin (*P*=0.9). A significant association was observed between high pretreatment serum folate and worse toxicity (*n*=38, *P*=0.02, CI: 1.0–1.2). On further analysis, elevated levels of pretreatment serum folate were associated with a higher incidence of grade 2/3 diarrhoea (*P*=0.001) and grade 2/3 nausea and vomiting (*P*=0.032) compared to grade 0/1 toxicity. For serum folate levels less than 17.96 nmol l^−1^, no significant toxicity was seen. When assessing for potential predictors of toxicity over the entire treatment period, serum folate remains significant (*P*=0.04, CI: 1.0–1.2).

## DISCUSSION

In the current study, we demonstrated that fixed dose capecitabine can be safely and effectively used as an alternative to conventional BSA-corrected dosing strategy in the treatment of metastatic colorectal cancer. Furthermore, we have shown that a raised serum folate level may be predictive of worse toxicity, in particular diarrhoea, nausea and vomiting.

With respect to efficacy, the overall response rate was 28%, time to disease progression was 4.9 months and median overall survival was 11.2 months. These results are similar to the combined results of phase III studies of the currently recommended BSA-calculated dosage schedule for capecitabine, which recruited over 1200 patients: overall response rate of 26%, time to disease progression of 4.6 months and overall survival of 12.9 months (Hoff *et al*, 2001; Van Cutsem *et al*, 2001, 2004). In comparing these results, it is essential to compare and contrast the clinical and demographic characteristics of the patient populations treated. Patients in the phase III studies received capecitabine exclusively as first-line treatment for metastatic disease, and 24% had received prior 5-FU-based adjuvant treatment. Similarly in our study, all but two patients were treated first line (96%) and the same proportion (24%) had been treated with prior 5-FU-based adjuvant therapy. However, the median age of the patients in our study population was considerably higher than in the phase III studies (72 *vs* 64 years, respectively). Furthermore, our results concur with the efficacy results of the BSA-calculated dose schedule of capecitabine in a similarly elderly population (median 75 years). In a study reported by Feliu *et al* (2005), 51 patients were treated with capecitabine first line for advanced/metastatic colorectal cancer. The response rate was 27% with an overall median survival of 11 months.

Overall fixed-dose capecitabine was well tolerated. As anticipated, diarrhoea and HFS were the predominant adverse effects. The majority of adverse events resolved with treatment delay, while a single dose reduction was required in 29% of patients and multiple reductions in only 9%. In total, 15% of patients ceased treatment because of adverse events and this was mainly due to gastrointestinal events. There were no treatment-related grade 4 adverse events or deaths.

The toxicity data reported in this study are comparable to that of the combined safety analysis of the two-phase III studies of BSA-calculated dose schedule of capecitabine (Cassidy *et al*, 2002). The rates of grade 3/4 adverse events in the combined analysis as compared to the current study of fixed-dose capecitabine were: diarrhoea (12 *vs* 9%), HFS (17 *vs* 11%), nausea and vomiting (2 *vs* 2%) and stomatitis (2 *vs* 0%). Therefore, toxicity appeared to be comparable in those receiving fixed-dose capecitabine.

In terms of exposure to capecitabine, patients received a median 6.5 treatment cycles. The total dose of capecitabine administered during the first cycle was 56 000 mg, which is 12% less than would have been administered using the currently recommended BSA-calculated dose schedule, that is, 63 350 mg. The lower total dose with the fixed-dose schedule might be expected to bias towards reduced efficacy and improved safety, but this was not found. Dose reduction during subsequent cycles is relatively common with either schedule: dose reduction occurred in 34% of patients receiving BSA-adjusted dosing with capecitabine in the phase III trials compared with a similar percentage (38%) in our study of fixed-dose capecitabine (Cassidy *et al*, 2002).

A number of studies have sought to identify patient characteristics and laboratory parameters that might predict for 5-FU toxicity. The second aspect of this trial was an assessment of likely predictors of toxicity in patients treated with capecitabine. A number of parameters were considered based on previous work with antifolate drugs (Calvert, 2002; Niyikiza *et al*, 2002). These included vitamin B12, serum and red cell folate and homocysteine. Based on previously published work, it was anticipated that there would not be a high incidence of grades 3 and 4 toxicity. Therefore, we planned to group grades 0 and 1 toxicity and grades 2 and 3 toxicity for analysis. Grade 2 mucositis, diarrhoea and HFS are significant toxicities, impacting negatively on quality of life and often necessitate treatment interruption, and predict for greater toxicity with subsequent courses, especially HFS. We found no significant relationship between toxicity and baseline values for serum vitamin B12, red cell folate or homocysteine. A significant relationship, however, was observed between high pretreatment concentrations of serum folate, and a higher incidence of grade 2/3 toxicity during cycle 1 (*P*=0.02). This association remained significant for the entire treatment period (*P*=0.04). In particular, a higher incidence of grade 2/3 diarrhoea and nausea and vomiting was seen during cycle 1. These results are contrary to studies in the antimetabolites, in which low levels of serum folate, as indicated by a raised serum homocysteine level, were associated with worse toxicity (Bunn *et al*, 2001; Niyikiza *et al*, 2002). This difference may be explained by the different mechanism of action of the antifolates. The antifolates compete with naturally occurring folate for active moieties on folate-dependent enzymes. Depletion in the level of intracellular folate will therefore lead to increased toxicity. Furthermore, the level of intracellular folate feeds back to inhibit the polyglutamation of antifolates, limiting toxicity. It could be hypothesised that increased serum folate results in an increase in the reduced folate pool and hence increased TS inhibition, increased drug activity and, therefore, increased toxicity. This, however, remains to be further elucidated. Furthermore, as only 36 folate samples were available for analysis, these results need to be interpreted with caution and a larger study needs to be conducted to confirm these results.

It is unclear why serum folate and not red cell folate should be correlated with worse toxicity. Serum folate levels are indicative of the immediate folate pool and changes with dietary intake. Red cell folate is the polyglutamated form of folate and is a measure of intracellular folate and therefore does not fluctuate on a daily basis. We could speculate then that because of the addition of a glutamate residue, red cell folate may perhaps be less mobile and therefore be unable to participate in enzymatic reactions, while serum folate may be more mobile.

We conclude that fixed-dose capecitabine is both an effective and well-tolerated treatment for patients with metastatic colorectal cancer. It allows simplification and ease of dosing, not only from the point of dose calculation but also because the initial dose (and any possible dose reductions) can be simply made up using the 500-mg tablet. This provides convenience for the patient and avoids potential confusion that might lead to miss-dosing by confusion between 500 and 150-mg tablets. The current results also suggest that a high pretreatment concentration of serum folate may be predictive of increased toxicity from capecitabine. This observation requires further investigation.

## Figures and Tables

**Table 1:** Demographic and clinical characteristics (*N*=55)

**Parameter**	**No. of patients (%)**
Male	35 (64%)
Median age, years (range)	72 (42–86)
Median bodyweight, kg (range)	71 (44.3–117.8)
	
*Performance status*	
0	20 (36%)
1	32 (58%)
2	3 (5%)
	
*Primary tumour site*	
Colon	39 (71%)
Rectal	15 (27%)
Unknown	1 (2%)
	
*No. of metastatic sites*	
1	30 (55%)
2	15 (27%)
⩾3	10 (18%)
	
Prior adjuvant chemotherapy	13 (24%)
Prior chemotherapy for metastatic disease	2 (4%)
Prior pelvic radiotherapy	6 (11%)

**Table 2:** Radiological response in the evaluable population (*N*=53)

**Response**	**No. of patients (%)**
Complete response	0
Partial response	15 (28)
Stable disease	17 (32)
Progressive disease	21 (40)

**Table 3:** Adverse events in the total population (*N*=55)

	**% of patients**	
	**Grade 2**	**Grade 3**
Fatigue	25	2
Hand–foot syndrome	11	11
Stomatitis	15	0
Diarrhoea	25	9
Nausea and vomiting	9	2
Anaemia	13	0
Liver toxicity	4	0

**Table 4:** Baseline laboratory test values

**Parameter (no. of patients with available results)**	**Median (range)**
Creatinine clearance, ml/min (*n*=55)	75.41 (45.12–165.1)
Red cell folate, nmol/l (*n*=28)	633.5 (358–1841)
Serum folate, nmol/l (*n*=38)	16.65 (6.6–45.4)
Albumin, g/l (*n*=51)	39 (29–47)
Vitamin B12, pmol/l (*n*=38)	283 (187–830)
Homocysteine, *μ*mol/l (*n*=36)	12 (8–47)
CEA (*n*=50)	52.8 (1.7–3225)

CEA=carcinoembryonic antigen.

## References

[bib1] Ardalan B, Flores MR (1995) A new complication of chemotherapy administered via permanent indwelling central venous catheter. Cancer 75: 2165–2168 7697607 10.1002/1097-0142(19950415)75:8<2165::aid-cncr2820750821>3.0.co;2-w

[bib2] Blum JL, Jones SE, Buzdar AU, LoRusso PM, Kuter I, Vogel C, Osterwalder B, Burger HU, Brown CS, Griffin T (1999) Multicenter phase II study of capecitabine in paclitaxel-refractory metastatic breast cancer. J Clin Oncol 17: 485–493 10080589 10.1200/JCO.1999.17.2.485

[bib3] Bunn P, Paoletti P, Niyikiza C, Rusthoven J, Nelson K, Hanauske AR, Stabler S, Calvert AH, Allen R (2001) Vitamin B12 and folate reduce toxicity of Alimta™ (pemetrexed disodium, LY231514, MTA), a novel antifolate/antimetabolite. Proc Am Soc Clin Oncol 20: 76a (abstr 300)

[bib4] Calvert H (2002) Folate status and the safety profile of antifolates. Semin Oncol 29: 3–7 10.1016/s0093-7754(02)70209-1 12023786

[bib5] Cassidy J, Twelves C, Cameron D, Steward W, O'Byrne K, Jodrell D, Banken L, Goggin T, Jones D, Roos B, Bush E, Weidekamm E, Reigner B (1999) Bioequivalence of two tablet formulations of capecitabine and exploration of age, gender, body surface area, and creatinine clearance as factors influencing systemic exposure in cancer patients. Cancer Chemother Pharmacol 44: 453–460 10550565 10.1007/s002800051118

[bib6] Cassidy J, Twelves C, Van Cutsem E, Hoff P, Bajetta E, Boyer M, Bugat R, Burger U, Garin A, Graeven U, McKendric J, Maroun J, Marshall J, Osterwalder B, Perez-Manga G, Rosso R, Rougier P, Schilsky RL (2002) First-line oral capecitabine therapy in metastatic colorectal cancer: a favorable safety profile compared with intravenous 5-fluorouracil/leucovorin. Ann Oncol 13: 566–575 12056707 10.1093/annonc/mdf089

[bib7] de Gramont A, Figer A, Seymour M, Homerin M, Hmissi A, Cassidy J, Boni C, Cortes-Funes H, Cervantes A, Freyer G, Papamichael D, Le Bail N, Louvet C, Hendler D, de Braud F, Wilson C, Morvan F, Bonetti A (2000) Leucovorin and fluorouracil with or without oxaliplatin as first-line treatment in advanced colorectal cancer. J Clin Oncol 18: 2938–2947 10944126 10.1200/JCO.2000.18.16.2938

[bib8] Douillard JY, Cunningham D, Roth AD (2000) Irinotecan combined with fluorouracil compared with fluorouracil alone as first-line treatment for metastatic colorectal cancer: a multicentre randomised trial. Lancet 355: 1041–1047 10744089 10.1016/s0140-6736(00)02034-1

[bib9] Feliu J, Escudero P, Llosa F, Bolanos M, Vicent JM, Yubero A, Sanz-Lacalle JJ, Lopez R, Lopez-Gomez L, Casado E, Gomez-Reina MJ, Gonzalez-Baron M (2005) Capecitabine as first-line treatment for patients older than 70 years with metastatic colorectal cancer: an oncopaz cooperative group study. J Clin Oncol 23: 3104–3111 15860870 10.1200/JCO.2005.06.035

[bib10] Giacchetti S, Perpoint B, Zidani R, Le Bail N, Faggiuolo R, Focan C, Chollet P, Llory JF, Letourneau Y, Coudert B, Bertheaut-Cvitkovic F, Larregain-Fournier D, Le Rol A, Walter S, Adam R, Misset JL, Levi F (2000) Phase III multicenter randomized trial of oxaliplatin added to chronomodulated fluorouracil–leucovorin as first-line treatment of metastatic colorectal cancer. J Clin Oncol 18: 136–147 10623704 10.1200/JCO.2000.18.1.136

[bib11] Gurney H (1996) Dose calculation of anticancer drugs: a review of the current practice and introduction of an alternative. J Clin Oncol 14: 2590–2611 8823340 10.1200/JCO.1996.14.9.2590

[bib12] Hoff PM, Ansari R, Batist G, Cox J, Kocha W, Kuperminc M, Maroun J, Walde D, Weaver C, Harrison E, Burger HU, Osterwalder B, Wong AO, Wong R (2001) Comparison of oral capecitabine *vs* intravenous fluorouracil plus leucovorin as first-line treatment in 605 patients with metastatic colorectal cancer: results of a randomized phase III study. J Clin Oncol 19: 2282–2292 11304782 10.1200/JCO.2001.19.8.2282

[bib13] Kolesar JM, Johnson CL, Freeberg BL, Berlin JD, Schiller JH (1999) Warfarin-5-FU interaction – a consecutive case series. Pharmacotherapy 19: 1445–1449 10600095 10.1592/phco.19.18.1445.30897

[bib14] Midgley R, Kerr D (1999) Colorectal cancer. Lancet 353: 391–399 9950460 10.1016/S0140-6736(98)07127-X

[bib15] Miwa M, Ura M, Nishida M, Sawada N, Ishikawa T, Mori K, Shimma N, Umeda I, Ishitsuka H (1998) Design of a novel oral fluoropyrimidine carbamate, capecitabine, which generates 5-fluorouracil selectively in tumours by enzymes concentrated in human liver and cancer tissue. Eur J Cancer 34: 1274–1281 9849491 10.1016/s0959-8049(98)00058-6

[bib16] National Cancer Institute Common toxicity criteria, v 2.0. http://ctep.cancer.gov/forms/CTCManual_v4_10-4-99.pdf 15332541

[bib17] Niyikiza C, Baker SD, Seitz DE, Walling JM, Nelson K, Rusthoven JJ, Stabler SP, Paoletti P, Calvert AH, Allen RH (2002) Homocysteine and methylmalonic acid: markers to predict and avoid toxicity from pemetrexed therapy. Mol Cancer Ther 1: 545–552 12479273

[bib18] Pinedo HM, Peters GF (1988) Fluorouracil: biochemistry and pharmacology. J Clin Oncol 6: 1653–1664 3049954 10.1200/JCO.1988.6.10.1653

[bib19] Reigner B, Blesch K, Weidekamm E (2001) Clinical pharmacokinetics of capecitabine. Clin Pharmacokinet 40: 85–104 11286326 10.2165/00003088-200140020-00002

[bib20] Saltz LB, Cox JV, Blanke C (2000) Irinotecan plus fluorouracil and leucovorin for metastatic colorectal cancer. Irinotecan Study Group. N Engl J Med 343: 905–914 11006366 10.1056/NEJM200009283431302

[bib21] Sawyer M, Ratain MJ (2001) Body surface area as a determinant of pharmacokinetics and drug dosing. Invest New Drugs 19: 171–177 11392451 10.1023/a:1010639201787

[bib22] Schmoll HJ (1996) Development of treatment for advanced colorectal cancer: infusional 5-FU and the role of new agents. Eur J Cancer 32A(Suppl 5): S18–S22 10.1016/s0959-8049(96)00335-8 8958038

[bib23] Schmoll HJ, Buchele T, Grothey A, Dempke W (1999) Where do we stand with 5-fluorouracil? Semin Oncol 26: 589–605 10606252

[bib24] Therasse P, Arbuck SG, Eisenhauer EA, Wanders J, Kaplan RS, Rubinstein L, Verweij J, Van Glabbeke M, van Oosterom AT, Christian MC, Gwyther SG (2000) New guidelines to evaluate the response to treatment in solid tumors. European Organization for Research and Treatment of Cancer, National Cancer Institute of the United States, National Cancer Institute of Canada. J Natl Cancer Inst 92: 205–216 10655437 10.1093/jnci/92.3.205

[bib25] Van Cutsem E, Hoff PM, Harper P, Bukowski RM, Cunningham D, Dufour P, Graeven U, Lokich J, Madajewicz S, Maroun JA, Marshall JL, Mitchell EP, Perez-Manga G, Rougier P, Schmiegel W, Schoelmerich J, Sobrero A, Schilsky RL (2004) Oral capecitabine *vs* intravenous 5-fluorouracil and leucovorin: integrated efficacy data and novel analyses from two large, randomised, phase III trials. Br J Cancer 90: 1190–1197 15026800 10.1038/sj.bjc.6601676 PMC2409640

[bib26] Van Cutsem E, Twelves C, Cassidy J, Allman D, Bajetta E, Boyer M, Bugat R, Findlay M, Frings S, Jahn M, McKendrick J, Osterwalder B, Perez-Manga G, Rosso R, Rougier P, Schmiegel WH, Seitz JF, Thompson P, Vieitez JM, Weitzel C, Harper P (2001) Oral capecitabine compared with intravenous fluorouracil plus leucovorin in patients with metastatic colorectal cancer: results of a large phase III study. J Clin Oncol 19: 4097–4106 11689577 10.1200/JCO.2001.19.21.4097

[bib27] van der Wilt CL, Backus HH, Smid K, Comijn L, Veerman G, Wouters D, Voorn DA, Priest DG, Bunni MA, Mitchell F, Jackman AL, Jansen G, Peters GJ (2001) Modulation of both endogenous folates and thymidine enhance the therapeutic efficacy of thymidylate synthase inhibitors. Cancer Res 61: 3675–3681 11325838

[bib28] Verso M, Agnelli G (2003) Venous thromboembolism associated with long-term use of central venous catheters in cancer patients. J Clin Oncol 21: 3665–3675 14512399 10.1200/JCO.2003.08.008

